# Factors affecting the measurements of peripheral oxygen saturation values in healthy young adults

**DOI:** 10.1515/med-2025-1219

**Published:** 2025-06-12

**Authors:** Oktay Uysal, Dilek Yılmaz

**Affiliations:** Yalova Training and Research Hospital, Intensive Care Unit, Centre/Yalova, 77200, Turkey; Department of Nursing, Faculty of Health Sciences, Bursa Uludağ University, Nilüfer/Bursa, 16059, Turkey

**Keywords:** body positions, healthy individuals, peripheral oxygen saturation, oxygenation, pulse oximeter

## Abstract

**Objectives:**

The aim of the study was to examine peripheral oxygen saturation values in the index and middle fingers of the dominant hand in healthy young adults measured while lying in a supine position or sitting straight up.

**Methods:**

This study was a non-randomized and non-controlled, quasi-experimental repeated measures study. It was conducted with 200 healthy young adults in Turkey. A pulse oximeter was located at the same time on the index and middle fingers of the active hand of each participant while sitting upright, and after being kept there for 1 min, the results of the measurements were recorded. Later, the participants were brought into a supine position, and after 10 min of rest, measurements with a pulse oximeter placed on the index and middle fingers of the active hand were repeated in the same way.

**Results:**

The mean age of the participants was 20.4 ± 1.8 years; 76.5% were female, and their mean body mass index was 22.5 ± 3.6 kg/m^2^. It was found that when the individuals were sitting upright, the peripheral oxygen saturation values measured from the middle finger were significantly higher than the peripheral oxygen saturation values measured from the middle finger in the supine position (*p* = 0.003). It was found that the peripheral oxygen saturation values of female participants measured from the index finger in the upright sitting position and from the index and middle fingers in the supine position were significantly higher than the saturation values of male participants (*p* = 0.018, *p* < 0.001, *p* = 0.001, respectively). In addition, it was found that the peripheral oxygen saturation values measured from the index and middle fingers of underweight individuals in the sitting position and from the index and middle fingers in the supine position were significantly higher than the saturation values of overweight individuals (*p* = 0.021, *p* = 0.006, *p* = 0.001, respectively).

**Conclusion:**

In the conclusions of this study, it was found that the highest oxygen saturation value of the young adults was measured from the middle finger when they were in the upright sitting position. It was also found that the variables of gender and body mass index significantly affected the peripheral oxygen saturation value.

## 1 Introduction

In various clinical situations, taking account of pulse oximetry or peripheral oxygen saturation measurement is a necessary and important parameter [1]. Peripheral oxygen saturation shows the oxygen saturation of hemoglobin (Hg) in the erythrocytes, i.e., the rate of attachment of oxygen to Hg [2,3]. It is expressed with a percentage value as SpO_2_. In healthy individuals, the SpO_2_ value varies between 95 and 100% [4,5]. Peripheral oxygen saturation is an important parameter used in assessing the necessity of oxygen treatment and the follow-up and effectiveness of treatment, in the routine monitoring of patients carried out in treatment and care in intensive care units, in patient monitoring in the operating theater, and especially in the evaluation of hypoxia [6–9].

Technological advances have enabled the frequent measurement of peripheral oxygen saturation levels with pulse oximeter devices, which are important in evaluating vital signs in the practice of basic clinic today [2,10,11]. The pulse oximeter is a non-invasive means of giving information on oxygen saturation by measuring changes in light absorption relating to arterial oxyhemoglobin by illuminating the skin using two wavelengths of light [12,13]. A pulse oximeter measures oxygen saturation by comparing the light absorption of oxyhemoglobin and deoxyhemoglobin by passing light at two wavelengths through a bed of tissue [14]. This technology has been widespread in clinical practice since the 1980s, and is used to give information on patient care and to improve results [2]. In this way, a pulse oximeter enables the prevention of arterial hypoxemia by providing results on peripheral oxygen saturation and the pulse in a short time, and is widely accepted as a standard of care [8,14–16]. It has been emphasized that measurement of peripheral oxygen saturation with a pulse oximeter is of great importance in the evaluation of vital signs in basic nursing practices [2,7,17]. In evaluation of this kind, the pulse oximeter has advantages such as being able to continuously monitor saturation, being easy to interpret, and being easy to use in most situations [16].

Positioning patients under the health professional’s responsibility is an independent intervention. Although it is important in patients with cardiovascular or cardiopulmonary dysfunction or oxygenation problems, there is insufficient evidence to recommend a certain position [18,19]. Health professionals frequently perform position change in the clinical field with the aim of reducing pressure as much as possible in patients, increasing their comfort and helping secretion from the liver, to preserve the patients’ health and to contribute to their recovery [7,19,20]. Positioning patients correctly increases liver volume, reduces heart rate, and improves ventilation and perfusion, thus affecting the level of blood oxygen saturation. The optimum level of oxygenation varies in connection with ventilation and perfusion [18,21].

The reliability of peripheral oxygen saturation read on the pulse oximeter can be affected by the individual who is being measured, the person making the measurement, and environmental factors [7,11]. It is reported in the literature that peripheral oxygen saturation measured with the pulse oximeter can be affected by different body positions [20–22], different parts of the body where the measurement is made [15], and different fingers which are measured [1,14,23–26].

In a current study, it was found that the peripheral oxygen saturation value was measured as significantly the highest in the middle finger, and that there was no difference in peripheral oxygen saturation between the fingers of the dominant and non-dominant hands [25]. It was concluded in a study by Swain et al. [27] that the peripheral oxygen saturation value was measured as significantly the highest in the middle fingers. In another study, it was shown that the peripheral oxygen saturation value could be affected by different body positions, with the highest value in the upright sitting position, and the lowest value in the supine position [20]. In another study, it was found that the highest peripheral oxygenation value was measured in the half-sitting position [21].

Discussion continues on the question of the best and most reliable position and choice of finger for the measurement of peripheral oxygen saturation. In a scan of the literature, very few studies were found examining together the effects on peripheral oxygen saturation values of different positions and fingers. Therefore, correct measurement of peripheral oxygen saturation and a good knowledge of the factors affecting it are of great importance for effective treatment and care. The aim of this study was to examine peripheral oxygen saturation values in the index and middle fingers of the dominant hand in healthy young adults measured while lying in a supine position or sitting straight up.

### 1.1 Research hypotheses

The hypotheses developed according to the aims of the research were as follows:

**Hypothesis 1:** In healthy young individuals, different body positions have an effect on peripheral oxygen saturation.

**Hypothesis 2:** In healthy young individuals, measurement on different fingers has an effect on peripheral oxygen saturation.

## 2 Materials and methods

### 2.1 Study design

This study was a non-randomized and non-controlled, quasi-experimental repeated measures study. The Strengthening the Transparent Reporting of Evaluations with Nonrandomized Designs (TREND) checklist was used as the reporting guideline for this study.

### 2.2 Study setting

The research was conducted between January and May 2024 in the Professional Skills Application Laboratory of the Health Sciences Faculty of a government university in the Marmara region of Turkey. This laboratory has an area of 127 m^2^, and consists of two separate rooms.

### 2.3 Participants and sampling

The research sample consisted of 200 healthy young individuals studying at the Health Sciences Faculty, who carried the criteria for inclusion in the research and who voluntarily agreed to participate. The criteria for the inclusion of individuals in the research were determined as follows: being aged between 18 and 35, having no hindrance in taking up any position, not having any acute or chronic disease, not being pregnant, not having artificial fingernails, nail varnish or henna on the hands, having a body temperature measured on the forehead of 36.0–37.9°C, having a pain intensity of 0 measured on the Visual Analog Scale before measurement, and volunteering to participate in the research.

The criteria for exclusion from the research were not volunteering to participate in the research, having diabetes mellitus, hypertension, hypotension, heart failure, coronary artery disease or blood coagulation disorders, having anemia or hemoglobinopathy, taking active exercise in the previous 30 min, eating at least 1 h before measurements were taken, and a history of smoking or currently smoking.

**Figure 1:**
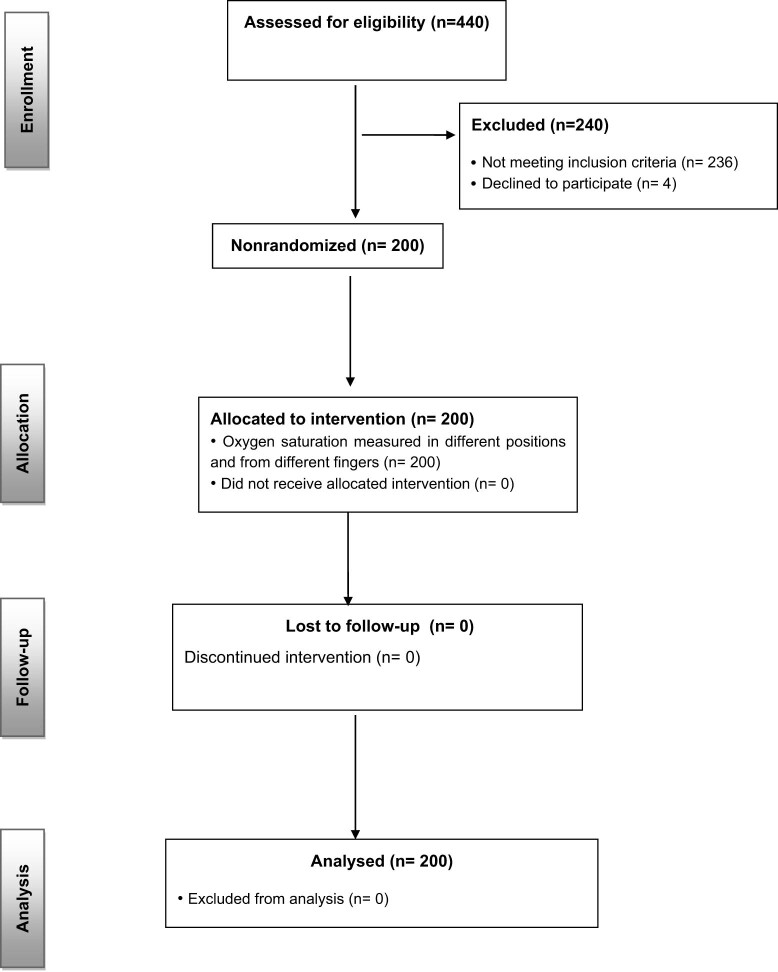
Flow chart of the TREND shows the number of the participants through each stage of the study.

The sample size was determined statistically by power analysis. Power analysis was performed using the program G*Power 3.1.9.6 (Kiel University, Germany). The study by Ceylan et al. [20] was taken as a reference in determining the sample size. In determining the effect size for oxygen saturation as 15% and considering a correlation level between measures for a 0.05 significance level and 80% power, the number of participants to be included in the study was calculated as 150. Taking into account possible data losses during the data collection process, a total of 200 individuals were included in the research. The data of all 200 individuals were collected completely, and no data were lost during the research. Figure 1 shows the research flow chart according to TREND.

### 2.4 Data collection instruments

An individual description form and a peripheral oxygen saturation form were used to collect research data.

#### 2.4.1 Individual description form

This form, developed to determine the descriptive information of the individuals, collected information on the person’s age, gender, height, weight, body mass index (BMI), body temperature, and dominant or active hand (left/right). BMI was classified according to the WHO classification: below 18.50 kg/m^2^ was classified as underweight, 18.5–24.99 kg/m^2^ as normal weight, 25–29.99 kg/m^2^ as overweight, and 30 kg/m^2^ or more as obese [28].

#### 2.4.2 Peripheral oxygen saturation monitoring form

This form was used to record the individuals’ peripheral oxygen saturation values measured from the index and middle fingers of the active hand when sitting upright and in the supine position.

### 2.5 Data collection

The research data were collected in the Professional Skills Application Laboratory. Before measurements were taken, the laboratory doors were kept closed to ensure room temperature, and care was taken to maintain the temperature while measurements were being made. A room thermometer was used for this purpose. Because daylight might affect the measured values, the laboratory’s window blinds were completely closed, and the light of the environment was used.

Considering that a full stomach might change lung capacity and affect measurement results, measurements were made between 10:00 and 12:00, before the midday meal. The individuals were asked when they had last eaten, and attention was paid to whether they had eaten at least 1 h before measurements were made.

Individuals who were included in the research were informed by the researcher about the aim of the research and the methods used, and the necessary legal approval was obtained. After that measurements were made of their height, using a portable stadiometer, and their weight, using a digital scale of ±0.1 kg sensitivity. After that, their body temperature was measured from the forehead with an infrared thermometer. All of these measured values were recorded on the individual description form.

Great care was taken to the research data being collected in an environment of infectious disease. While the study was being conducted, the necessary measures were taken to prevent the individuals from affecting each other, such as not speaking to each other and not being in contact.

All those who were included in the research were allowed to rest for 5 min before measurements were made [24–27]. After that, the individuals were made to sit upright on the bed in the laboratory, and at the same time a pulse oximeter (Life Net Medicel KE-6004- Turkey) was located on the index and middle fingers of their active hand. Because it was reported in some studies that the measured peripheral oxygen saturation was higher in the fingers of the active hand [14,24], measurements were made in this study only from the fingers of the participants’ active hands.

In order to prevent measurement errors which could arise in relation to hand movement, the individuals were asked to keep their hands still during the procedure. The pulse oximeter was located at the same time on the index and middle fingers of the active hand. After leaving it on the two fingers for 1 min [6,25,29], stable values seen for at least 3 s on the pulse oximeter screen [27] were recorded on the form.

After the measurements made in the upright sitting position were recorded, the bed head was immediately brought to zero degrees, and the individual was placed in the supine position. While in this position, a thin pillow was located under the individual’s head [20]. It is reported in the literature that after a position change, fluctuations in pressure may occur [5,30]. Keeping in mind the probability that this might change the individuals’ hemodynamic parameters, the individuals we allowed to rest for 10 min in this position. After this 10 min rest, the peripheral oxygen saturation measurement procedure previously used in the sitting position was repeated on the same hand and fingers. All measurements were made by the same researcher. Recording on the data collection form of the individuals’ peripheral oxygen saturation values was performed by a person who was independent of the research (a master’s student in the field of nursing who was not taking part in the research).

### 2.6 Data analysis

Statistical analysis of the research data was performed with the statistics package IBM SPSS 28.0 (IBM Corp. released 2021. IBM SPSS Statistics for Windows, Version 28.0. Armonk, NY; IBM Corp.). The research data were tested with the Shapiro–Wilk test for conformity to normal distribution. Descriptive statistics were expressed with quantitative values, mean values, and standard deviations and qualitative data were expressed with frequencies and percentages. In comparing two independent groups, the Mann–Whitney *U*-test was used for data which was not normally distributed, and in comparing more than two groups, the Kruskal–Wallis test was used. When significance was found, the Bonferroni test, a multiple comparison test, was used. In the comparison of the measurement values of dependent groups, the Wilcoxon Signed Ranks Test was used. The level of statistical significance was taken as *p* < 0.05.


**Informed consent:** Oral and written approval was obtained from the individuals included in the research after they were informed about the aim and procedure of the research.
**Ethical approval:** This single-center, nonrandomized, quasi-experimental study was registered in the clinical trials database (https://clinicaltrials.gov; NCT06591403). Ethical approval to conduct the research was obtained from the Clinical Research Ethics Committee of the Medical Faculty of Bursa Uludağ University (Decision No: 2023-25/25). The research was conducted in conformity with the decisions of the Helsinki Declaration.

## 3 Results

The mean age of the participants was 20.4 ± 1.8 years; 76.5% were female, and their mean BMI was 22.5 ± 3.6 kg/m^2^. The measured mean body temperature of the participants before the peripheral oxygen saturation measurements was 36.42 ± 0.47°C, and 92% were found to use their right hand as their active hand (Table 1).

**Table 1:** Participants’ descriptive characteristics (*n* = 200)

Age: Mean (SD) years		20.4 (1.8)		
Body temperature: Mean (SD) (°C)		36.42 (0.47)		
BMI: Mean (SD) (kg/m^2^)		22.5 (3.6)		
			***n***	**(%)**
Gender	Female		153	76.5
	Male		47	23.5
Actively used hand	Right		184	92.0
	Left		16	8.0

**Figure 2:**
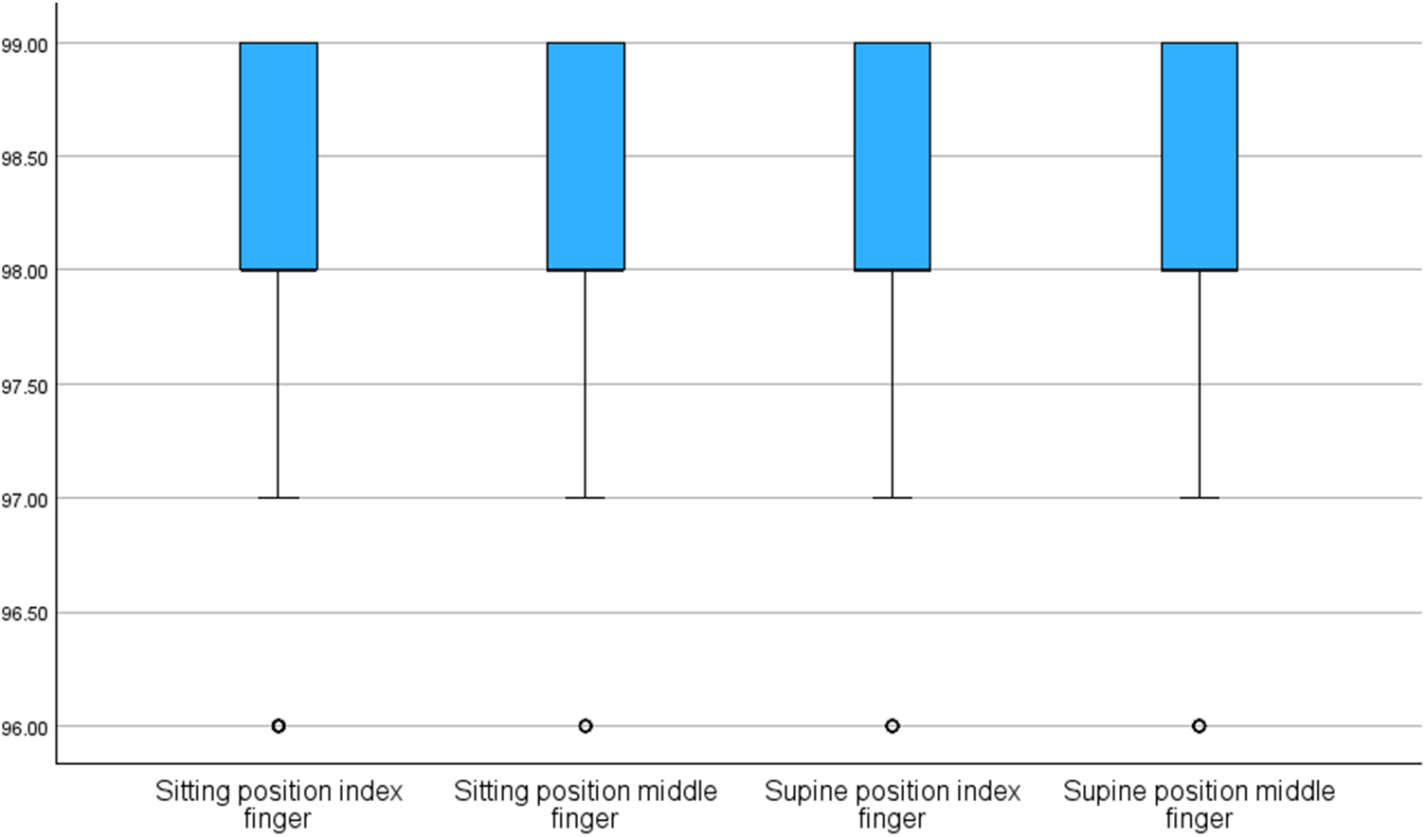
Results of participants’ peripheral oxygen saturation measurements made in sitting and supine positions from the index and middle fingers.

Table 2 and Figure 2 show the comparison of the peripheral oxygen saturation measurement results taken from the index and middle fingers in the sitting and supine positions. As a result of statistical analysis, it was found that the peripheral oxygen saturation value measured from the middle finger in the sitting position was significantly higher than the peripheral oxygen saturation value measured from the middle finger in the supine position (*p* = 0.003). No significant difference was found between peripheral oxygen saturation values measured from the index and middle fingers while the participants were in either a sitting or a supine position (*p* = 0.410, *p* = 0.949, respectively, Table 2).

**Table 2:** Comparison of results of participants’ peripheral oxygen saturation measurements made in sitting and supine positions from the index and middle fingers (*n* = 200)

	Sitting position		Supine position		
	Mean (SD)	Minimum–maximum	Mean (SD)	Minimum–maximum	Test value/*p*
Index finger	98.24 (0.84)	96.0–99.0	98.11 (0.83)	96.0–99.0	*Z* = −1.715, *p* = 0.086
Middle finger	98.30 (0.78)	96.0–99.0	98.11 (0.81*)	96.0–99.0	*Z* = −2.936, *p < 0.05
Test value/*p*	*Z* = −0.825; *p* = 0.410		*Z* = −0.064; *p* = 0.949		

Abbreviations: SD, standard deviation. Wilcoxon signed range test was used. *p < .05

Table 3 shows the results of peripheral oxygen saturation measurement according to the variables of participants’ gender and BMI. As a result of statistical analysis regarding the variable of gender, it was found that the peripheral oxygen saturation values of female participants measured from the index finger in the sitting position and from the index and middle fingers in the supine position were significantly higher than the peripheral oxygen saturation values of male participants (*p* = 0.018, *p* < 0.001, *p* = 0.001, respectively). A statistically significant difference was found between the variable of the participants’ BMI and their peripheral oxygen saturation values measured from the middle finger in a sitting position and from the index and middle fingers in a supine position. As a result of two-way comparisons made using the Bonferroni test to determine this difference, it was found that the peripheral oxygen saturation values of underweight individuals measured from the middle finger in the sitting position and in the supine position from the index and middle fingers were significantly higher than the peripheral oxygen saturation values of overweight individuals (*p* = 0.021, *p* = 0.006, *p* = 0.001, respectively). On the other hand, no statistically significant difference was found when comparing the peripheral oxygen saturation measurements obtained from the index and middle fingers of participants of the same gender and in the same BMI category in either position (*p* > 0.05, Table 3).

**Table 3:** Comparison of peripheral oxygen saturation values of participants by variables of gender and body mass index (*n* = 200)

			Sitting position	Sitting position	^c^Test value/*p*	Supine position	Supine position	^c^Test value/*p*
			Index finger	Middle finger		Index finger	Middle finger	
		n	Mean (SD)	Mean (SD)		Mean (SD)	Mean (SD)	
Gender	Female	153	98.30 (0.85)	98.33 (0.79)	*Z* = −0.521; *p =* 0.602	98.22 (0.84)	98.20 (0.78)	*Z* = −0.216; *p* = 0.829
	Male	47	98.06 (0.85)	98.25 (0.85)	*Z* = −0.849; *p* = 0.396	97.75 (0.85)	97.78 (0.83)	*Z* = −0.542; *p* = 0.588
	^a^Test value/*p*		*Z* = −2.356; *p* = 0.018	*Z* = −1.723; *p* = 0.085		*Z* = −1.523; *Z* = *Z* = −4.249; *p* < 0.001	*Z* = −3.217; *p* = 0.001	
BMI	Underweight	25	98.48 (0.50)	98.64 (0.56)*	*Z* = −1.265; *p* = 0.206	98.52 (0.65)*	98.52 (0.65)*	*Z* = 0.000; *p* = 1.000
	Normal	123	98.55 (0.85)	98.31 (0.78)	*Z* = −0.699; *p* = 0.484	98.11 (0.87)	98.20 (0.75)	*Z* = −0.622; *p* = 0.534
	Overweight	43	98.00 (0.95)	98.04 (0.84)	*Z* = −0.182; *p* = 0.856	97.83 (0.75)	97.69 (0.91)	*Z* = −0.862; *p* = 0.388
	Obese	9	98.25 (0.52)	98.33 (0.70)	*Z* = −1.414; *p* = 0.157	98.22 (0.66)	98.22 (0.83)	*Z* = 0.000; *p* = 1.000
	^b^Test value/*p*		*χ* ^2^ = 5.145; *p* = 0.161	*χ* ^2^ = 9.770; *p* = 0.021		*Z* = −1.523; *Z* = *χ* ^2^ = 12.401; *p* = 0.006	*χ* ^2^ = 17.028; *p* = 0.001	

Abbreviations: BMI, body mass index; SD, standard deviation. ^a^Mann–Whitney U test was used. ^b^Kruskal–Wallis test was used. Bonferroni test was used for pairwise comparisons. ^c^Wilcoxon signed rank test was used. Comparison with overweight: *p < 0.05.

## 4 Discussion

It has been emphasized that peripheral oxygen saturation measurement with a pulse oximeter is of great importance for the evaluation of vital signs in the field of basic health practices [2,10,17]. Evaluated from this angle, the pulse oximeter has advantages such as the ability to continuously measure saturation, to be easily interpreted, and to be easily used in most environments [16]. The reliability of the peripheral oxygen saturation read on the pulse oximeter may be affected by the person being measured, the person performing the measurement, or environmental factors [7]. Some studies have shown that body positions and the fingers measured have significant effects on peripheral oxygen saturation, and that these effects might in turn affect clinical decisions [20,22,25–27]. In this regard, the peripheral oxygen saturation values of the healthy young adults obtained from the index and middle fingers in the upright sitting and supine positions in this study were examined in detail while eliminating as much as possible the environmental and personal factors which might affect the measurement results. The research findings generally showed that similar measurement results were obtained from the middle and index fingers in upright sitting and supine positions, but that peripheral oxygen saturation values measured from the middle finger in the upright sitting position were significantly higher than the peripheral oxygen saturation values measured from the index finger while in the supine position. In this situation, it was seen that the mean peripheral oxygen saturation values of healthy young adults taken from the middle finger while in the upright sitting position was higher than the mean values of other measurement results. In the light of these results, hypotheses H_1_ and H_2_ were accepted. The results obtained from the research show that this situation should be taken into account in clinical evaluations. In addition, this data can provide an important instrument for understanding the effect of different positions and fingers in the measurement of peripheral oxygen saturation in clinical procedures and in patient care. In line with these results, when healthcare professionals are measuring peripheral oxygen saturation, as is frequently done in clinical areas, they may consider taking into account the tolerance of individuals by taking the measurement from the middle finger in the sitting position.

Examining the results of studies on the topic with healthy volunteers, it is seen that Basaranoglu et al. [24] obtained 370 peripheral oxygen saturation measurements from 37 volunteers. The highest peripheral oxygen saturation values were obtained from the middle finger. It was emphasized in the study that the peripheral oxygen saturation values obtained from the index finger and the thumb reflected the best result. In a study by Ceylan et al. [20], it was found that when healthy individuals were in different positions, there were significant differences in their peripheral oxygen saturation values, and that the mean peripheral oxygen saturation value measured in the upright sitting position was significantly higher than the mean peripheral oxygen saturation value measured in the supine position. Also, in a similar study by Tripathi et al. [26], it was concluded that the highest peripheral oxygen saturation value was measured on the middle finger. In a cross-sectional study by Sur and Kundu [25] with 100 volunteers aged 18–30, it was found that the highest peripheral oxygen saturation values were measured in the middle fingers. In a study by Swain et al. [27] conducted with 391 participants, peripheral oxygen saturation measurements were made from the fingers of both hands with participants in the upright sitting position. It was concluded that the highest peripheral oxygen saturation values in both hands were measured from the middle finger. In a study by Khan et al. [1] conducted with 200 volunteers, peripheral oxygen saturation measurements were made on the fingers of both hands with the participants in a sitting position, and it was found that the highest peripheral oxygen saturation value was recorded from the middle finger.

It is seen that the results of all of the studies mentioned above are similar to this study. From these results in the literature, it can be said that the highest peripheral oxygen saturation values obtained from healthy individuals were obtained from the middle finger in the upright sitting position. Also, some studies in the literature explain that a possible reason why the middle finger of the dominant hand has the highest perfusion is that blood flow is provided from both the radial and the ulnar arteries [14,24].

On the other hand, Sapra et al. [14] conducted a study examining the peripheral oxygen saturation values from different fingers of both hands of 96 healthy health workers in a hospital. As a result of the study, it was reported that the highest value was measured from the ring finger and the lowest from the thumb. In a study by Agrawal et al. [10] comparing the peripheral oxygen saturation measurement results between the two hands, a total of 930 measurements were obtained from 93 volunteers. It was concluded that in volunteers whose right hand was their active hand, the highest peripheral oxygen saturation value was measured from the left little finger. The results of these two studies are different from the results of this study. The differences between the studies may arise from differences in the environment where the measurements were made and from differences in the descriptive characteristics of the sample included in the study.

It is reported in the literature that the factor of gender may significantly affect peripheral oxygen saturation values [31–33]. It was found in this study that the peripheral oxygen saturation values measured from the index finger in the upright sitting position and from the index and middle fingers in the supine position of female participants were significantly higher than the values obtained from male participants. This finding shows that the peripheral oxygen saturation values of females are generally higher than those of males. In a study by Ceylan et al. [20] evaluating the peripheral oxygen saturation measurement results of healthy young individuals, it was found that the peripheral oxygen saturation values of female participants were significantly higher than those of male participants. It was concluded in a study by Levental et al. [33] examining whether there were differences in peripheral oxygen saturation between young healthy adult males and females that the peripheral oxygen saturation values measured in adult female was higher than in male adults. It was stated that this result may be related to the difference in hormonal status between the genders, especially progesterone, which stimulates respiration and increases oxygenation through chemoreceptors [33]. Therefore, a possible reason for increased peripheral oxygen saturation in females may be thought of as increased respiration rate in connection with hormonal change. However, it is reported in some studies that the peripheral oxygen saturation values of males were higher than those of females [31,34,36]. With the different results in the literature, it is seen that there is a need for more studies examining the differences between the genders in peripheral oxygen saturation values. Also, it must not be forgotten that the factor of gender is a variable which can affect an individual’s peripheral oxygen saturation measurement value.

In the present study, it was found that the peripheral oxygen saturation values measured from the middle fingers of underweight individuals in the upright sitting position and from the index and middle fingers in the supine position were significantly higher than the peripheral oxygen saturation values of overweight individuals. These findings show that the variable of BMI can affect the peripheral oxygen saturation measurement value. In a study by Ceylan et al. [20] conducted with healthy volunteers, it was found that peripheral oxygen saturation values measured in five different body positions were significantly high in participants whose mean BMI values were less than 25 kg/m^2^. It is stated in the literature that the changes in ventilation and oxygenation status that occur in overweight individuals increase the respiratory rate and oxygen consumption of individuals due to the increased metabolic needs of excess tissues [35,36]. Evaluated in this way, since the oxygen consumption of individuals with a high BMI may increase, the measured peripheral oxygen saturation value may be recorded as lower than that of individuals with a low BMI. The findings of this study support the results of the literature mentioned above. However, it is noticeable that no difference was seen in oxygenation between obese and underweight individuals participating in this study. This can be interpreted as the results not being significant because very few obese individuals participated in the study (*n* = 9).

This research has a number of limitations. It was conducted with healthy young individuals aged 18–35 who were known not to have an acute or chronic illness or anemia. For this reason, the results of the study cannot be generalized to children, old people, or those with any acute or chronic illness. A second limitation of the study is that the presence of anemia was taken into account dependent on the participants’ own statements. This may cover only those with a recent diagnosis of anemia, and therefore may bias the research findings. A further limitation is that peripheral oxygen saturation values were made only from the index and middle fingers of the individuals’ dominant hands. For this reason, it is recommended that research be conducted measuring peripheral oxygen saturation with different sample groups, in different positions, and taking account of the dominant/non-dominant use of the hand. Another limitation of the study is that the position in which the individuals were to be placed in the study was not determined randomly. A final limitation of the study is that female participants were not asked whether they were on their menstrual cycle when measurements were taken. There may be a possibility that the rising level of the hormone progesterone affected peripheral oxygen saturation. Taking account of all of these limitations, it is recommended that longitudinal and randomized controlled studies be conducted with a larger sample, in which individuals of different age groups, ethnic origins, and health problems are included. In these individuals, the presence or absence of anemia should be confirmed by medical records, menstrual cycles should be determined in female participants, environmental factors should be controlled more strictly, and peripheral oxygen saturation measurement results should be obtained from all fingers and both hands.

## 5 Conclusions

As a result of the research, it was concluded that the peripheral oxygen saturation values of healthy young adults measured when in the upright sitting or supine positions were not significantly affected by measurement from the index or middle fingers, but that the peripheral oxygen saturation value measured from the middle finger in the upright sitting position was significantly higher than the peripheral oxygen saturation value measured from the index finger in the supine position. Also, it was seen that the peripheral oxygen saturation values measured in both positions of female participants and those who were underweight according to their BMI were higher than males and overweight participants.

This study showed that the highest peripheral oxygen saturation value in healthy young adults was measured in the sitting position from the middle finger. This result obtained from the study shows that this should be taken into account in clinical evaluations. It was seen that there was no significance between peripheral oxygen saturation values measured in the supine position from the index and middle fingers. The research findings show the necessity of preferring the middle finger and the sitting position in the measurement of peripheral oxygen saturation. It is recommended that when health professionals are measuring peripheral oxygen saturation, they should take account of the fact that it can be affected by such variables as the patient’s position, the finger from which the measurement is taken, gender, and BMI. These results can provide an important criterion for understanding the effects of different positions and fingers in measuring peripheral oxygen saturation in clinical applications and in the conduct of patient care.

## Abbreviations

BMI: body mass index
Hg: hemoglobin
SpO_2_
: peripheral oxygen saturation
TREND: the Strengthening the Transparent Reporting of Evaluations with Nonrandomized Designs
